# Supplementary material for: Versatile Confocal Raman Imaging Microscope Built from Off-the-Shelf Opto-Mechanical Components
Source: Sensors (Basel). 2022 Dec 19;22(24):10013. doi: 10.3390/s222410013 (PMC9786121; doi:10.3390/s222410013)
Supplement: Supplementary file 1 [file sensors-22-10013-s001.zip › sensors-2050018_Supplementary WITH S.pdf]

SUPPLEMENTARY MATERIALS

Versatile confocal Raman imaging microscope built from off-the-shelf opto-mechanical components

Deseada Diaz Barrero[1], Genrich Zeller [2,*], Magnus Schlösser [2], Beate Bornschein[2] and Helmut H. Telle[1,*]

[1] Departamento de Química Física Aplicada, Universidad Autónoma de Madrid, Campus de Canto-blanco, 28049 Madrid, Spain

[2] Tritium Laboratory Karlsruhe (TLK), Institute for Astroparticle Physics (IAP), Karlsruhe Institute of Technology (KIT), Hermann-von-Helmholtz-Platz 1, 76344 Eggenstein-Leopoldshafen, Germany

* Correspondence: genrich.zeller@kit.edu; helmut@telle-online.eu

---

INDEX

S.A. **CRM system alignment and characterization**
S.A.1 Procedural details for the alignment of the CRM beam paths page S1
S.A.2 Axial focusing onto the sample surface page S3
S.A.3 Determination of the laser focal beam diameter by different means page S4
S.A.4 Automatic control for keeping samples in the laser focus page S7
S.A.5 Axial scanning page S8
S.A.6 The use of different pin holes and objectives page S10

S.B. **Measurements on selected samples**
S.B.1 Raster scans of graphene sheets and GFET sensing devices page S11
S.B.2 Comparison of confocal Raman raster maps vs SEM images page S13
S.B.3 Comparison of confocal Raman raster maps vs wide-field images page S14

S.A.1 – Procedural details for the alignment of the CRM beam paths

As stated in Section 3.1 of the main text, in our CRM a collimated laser beam is propagating via the dichroic beam splitter through the objective onto the target. Because of the delicate positioning of the beam splitter in its mounting cube, alignment of the incoming laser beam – and thereafter directing the reflection / emission from the target to the confocal pinhole in the Raman light collection path – constitutes a complex, though straight-forward task. A detailed recipe for this initial system alignment is given here.

Alignment of confocal Raman light path

In order to align the laser beam along the CRM-cage axis, first the objective was removed and the short cage structure was extended (see Figure S1, replicated here from Figure 1 in the main text, for convenience of the reader). With the aid of two cage alignment targets and manipulating the laser fiber collimator orientation using its $(x, y, \theta_x, \theta_y)$-kinematic mount, the laser beam was aligned as parallel and central as possible along the cage axis. After successful alignment, the cage extension is removed again.

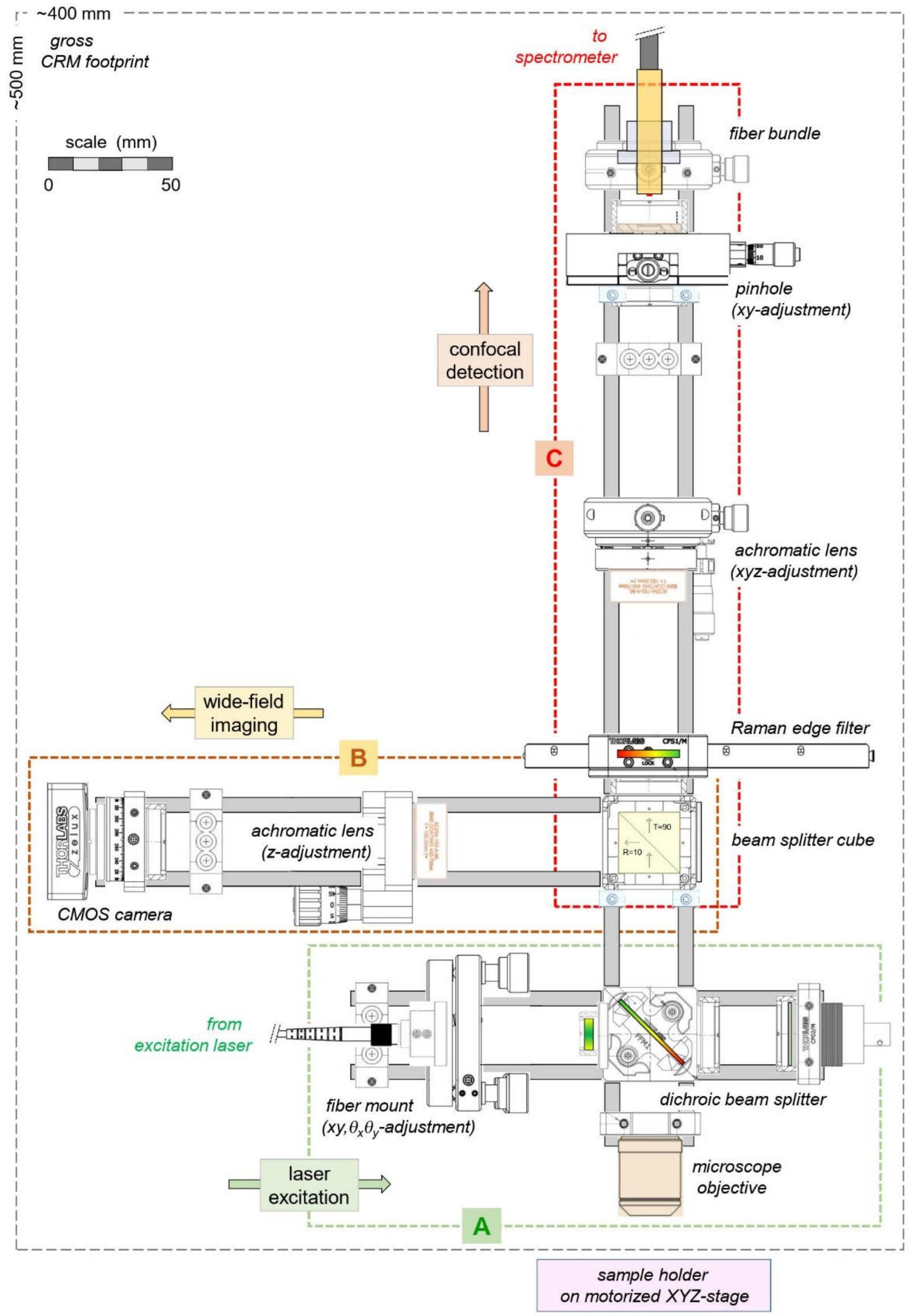

Figure S1. Overview of the confocal Raman microscope constructed from opto-mechanical cage system components and optical elements of the *Thorlabs* collection; note that the DPSS excitation laser, the Raman spectrometer and the motorized sample stage are not included here. The conceptual CRM groups are: A – excitation laser coupling and Raman light collection; B – the wide-field imaging arm; C – the confocal light collection arm. Key optical components are annotated, together with their spatial adjustment

A crystalline-Si SEM finder grid target (EM-Tec FG1, *Micro-to-Nano*), attached to the sample holder, plays a pivotal role in the task of fine alignment. The particular choice of target was made for two reasons. First, c-Si exhibits a high reflectivity of ~37 % at the laser wavelength λ_{laser} = 532 nm (see e.g. references [12,13]); thus it is suitable to act as a mirror for imaging of the laser focal spot onto the CMOS camera. Second, it has a strong Raman peak at 520.9 cm^{-1} [14] (since the early days of Raman spectroscopy with

lasers, this particular sample and its Raman peak are utilized for spectral calibration of Raman microscopes).

The Si-target, positioned roughly at the focus of the objective, reflects the laser beam back along the cage axis of the Raman detection arm. For this step, its Raman edge filter and achromatic lens were removed; for reference to the individual elements see Segment C in Figure S1. Note that, this alignment is slightly imperfect (with respect to “before” and “after” the 45° dichroic beam splitter), because of the minute beam displacement introduced by this optical filter. Note also that only ~0.5 % of the laser light passes through the beam splitter; however, this is sufficient to observe the laser beam travelling towards and through the confocal pinhole, as well as additional cage alignment targets before and after the pinhole. The pinhole is adjusted in its lateral xy-direction so it is positioned on axis. Thereafter, the removed optical elements are successively re-introduced into the CRM structure.

First, the objective is mounted again and the axial (z-direction) position of the Si-grid finder target is adjusted so that the beam propagating in the confocal Raman arm of the CRM is collimated again. Any lateral deviation from the axis, which might be introduced by imperfect cage centering of the objective, is compensated for by small adjustments to the laser fiber collimator orientation.

Second, once on-axis propagation is re-established, the focusing achromatic lens is inserted once more. Note that for ease of precise removal and re-insertion it is mounted in a precision magnetic quick-release mount (CXY1Q, *Thorlabs*). Its lateral xy- and axial z-positions are adjusted so that the light passes through the pinhole in focus.

Alignment and use of the wide field CMOS camera path

The coarse alignment of the axial position of the respective components in the wide-field imaging arm is performed by first accurately measuring the actual sensor position (relative to the entrance face of the CMOS camera), and then positioning the camera and lens in accordance with the focal distance, using e.g. a caliper.

By performing an axial (z-direction) scan of the sample, the surface position resulting in the maximum Raman signal is determined; this maximum constitutes the confocal “in-focus” condition.

Due to the aforementioned high reflectivity of silicon, a faint image of the laser spot from the Si-target surface is visible on the CMOS camera sensor. Now the axial position of the lens in the imaging arm is adjusted, such that the diameter of the laser spot image becomes minimized. At the end of the procedure, the maximal Raman signal corresponds to a minimum-sized laser spot image, i.e. the two arms are now perfectly matched with respect to each other.

References S.A1

[12] Aspnes, D.E.; Studna, A.A. Dielectric functions and optical parameters of Si, Ge, GaP, GaAs, GaSb, InP, InAs, and InSb from 1.5-6.0 eV, *Phys. Rev. B* **1983**, 27, 985-1009. https://doi.org/10.1103/PhysRevB.27.985

[13] Green, M.A.; Keevers, M. Optical properties of intrinsic silicon at 300 K. *Prog. Photovolt.* **1995**, 3, 189-192. https://doi.org/10.1002/pip.4670030303 Online access to numerical Si-reflectivity data: https://www.pveducation.org/pvcdrom/materials/optical-properties-of-silicon#footnote1_ciy5rpk Last accessed: Dec. 10, 2022.

[14] Deschaines, T.; Hodkiewicz, J.; Henson, P. Characterization of amorphous and microcrystalline silicon using Raman spectroscopy. Thermo Fisher Scientific, Madison (WI), USA. Application Note 51735, **2009**. Online access: https://assets.thermofisher.com/TFS-Assets/CAD/Application-Notes/D16998~.pdf Last accessed: Dec. 10, 2022.

S.A.2 – Axial focussing onto the sample surface

With the confocal and the wide-field imaging arms aligned and focally matched, the image of the laser spot on the CMOS camera can be used to determine the focus position of any sample, which exhibits decent reflectivity for laser light with $\lambda_{laser} = 532$ nm. This can be achieved without the need to perform an extended axial scan of the sample, which then is fitted to a Lorentz function to derive the maximum and Rayleigh depth (as outlined in Section A.5 below). Such a proposal for rapid (automated) focus control is discussed e.g. by Yazdanfar and co-workers [15]; note that, automated focus control is frequently used in commercial confocal laser microscopes.

In order to find the minimized laser spot image recorded by the CMOS camera, the sample is simply moved in axial z-direction around the guessed focus location. Using an intelligent algorithm, as suggested

in reference [RS4], the exact focal position can be determined from only a few images. Thus, it constitutes a much faster procedure than using the aforementioned axial Raman profiling.

Note however that, for transparent samples / substrates one has to ensure that the laser spot is indeed on the sample surface and not on the (metal) sample holder surface below. For transparent samples one can typically observe two focus positions with minimial laser spot size. The position closer to the CRM objective corresponds to the investigated sample surface (such as the graphene films discussed in Section A.5 below). Note also that for transparent samples it is recommended to roughen the surface of the sample holder, or blacken" it, in order to reduce the intensity of its laser light reflection.

References S.A2:

[15] Yazdanfar, J.; Kenny, K.B.; Tasimi, K.; Corwin, A.D.; Dixon, E.L.; Filkins, R.J. Simple and robust image-based autofocusing for digital microscopy. *Opt. Express* **2008**, 16, 8670-8677. https://doi.org/10.1364/OE.16.008670

S.A.3 – Determination of the laser focal beam diameter (FBD) by different means

Commonly, commercial laser beam profilers are used to capture and analyze the spatial intensity profile of a laser beam, in a plane transverse to the beam propagation path; devices based on 2D-array camera techniques with direct illumination of the camera sensor are the most popular. However, such instruments are not necessarily suitable if the beam diameter to be analyzed approaches the pixel size of the camera sensor, mostly of the order of 2-5 μm. For the spot size of a laser beam focused through a microscope objective this may become insufficient; in addition, it might be physically impossible to arrange the beam profiler in the focal plane of a microscope. Therefore, instead of direct beam profiling the (indirect) knife-edge technique is often utilized (see e.g. [25,26]): a slit or blade cuts the laser beam before detection by a power / intensity detector. By stepwise recording the integral intensity profile for a number of cuts, the original beam profile can be reconstructed, nowadays predominantly using algorithms developed for tomography.

Here we have applied a slight modification of the standard knife-edge method. Instead of measuring the detector response to the integrated laser intensity, partially obscured by the knife-edge, the laser beam moves across a sharp boundary with different response to the laser radiation on both sides. In particular, we have exploited the Raman signal response associated with different (molecular) sample compounds.

For the determination of the laser spot size we utilized two samples with distinct “edges” in their structure, namely (i) a silicon SEM finder grid substrate (*Micro to Nano* EM-Tec FG1); and (ii) a GFET sample (graphene square on a SiO_2 / Si substrate, *Graphenea* GFET-S10).

While primarily designed for SEM applications, the finder grid substrate is equally suitable for reflected light or Raman microscopy. The sample of size 12×12 mm^2 comprises a silicon monocrystal with <100> surface orientation onto which a 1×1 mm^2 grid of chromium (Cr) is deposited (nominally with thickness 75 nm and width 20 μm). The laser beam was focused onto the Si-substrate, minimizing its diameter as observed via the CMOS camera. Subsequently the sample position was changed in one direction (“horizontal” scan), in step increments of 0.5 (≡ 0.625 μm) across four consecutive grid lines, both in forward and backward direction.

The scan over one individual Cr-grid line is shown in the right data panel of Figure S1(a). According to the knife-edge treatment of a Gaussian laser beam with profile function

$$I(x,y) = I_0 \cdot \exp\left(-\frac{(x-x_0)^2+(y-y_0)^2}{w^2}\right) , \qquad \text{(eq.1)}$$

where I_0 is the peak intensity at the beam center (x_0, y_0) and w is the beam radius, measured at the 1/e point in the intensity distribution curve.

The normalized transmitted power is given by the integral expression (see e.g. reference [26])

$$P_{\mathrm{N}}(x) = \frac{\int_{-\infty}^{x}\int_{-\infty}^{\infty} I(x',y)\mathrm{d}y\mathrm{d}x'}{\int_{-\infty}^{\infty}\int_{-\infty}^{\infty} I(x',y)\mathrm{d}y\mathrm{d}x'} . \qquad \text{(eq.2)}$$

The evaluation of (eq.2) at each position x of the incremental knife-edge scan then yields

$$P_{\mathrm{N}}(x) = \frac{1}{2}\cdot\left[1+\mathrm{erf}\left(\frac{x-x_0}{w}\right)\right] \;, \qquad \text{(eq.3)}$$

where erf is the so-called error function. Note that the erf-function is equivalent to the cumulative distribution function of the standard normal distribution, normCDF (function built into Origin® / OriginPro® data analysis and graphing software).

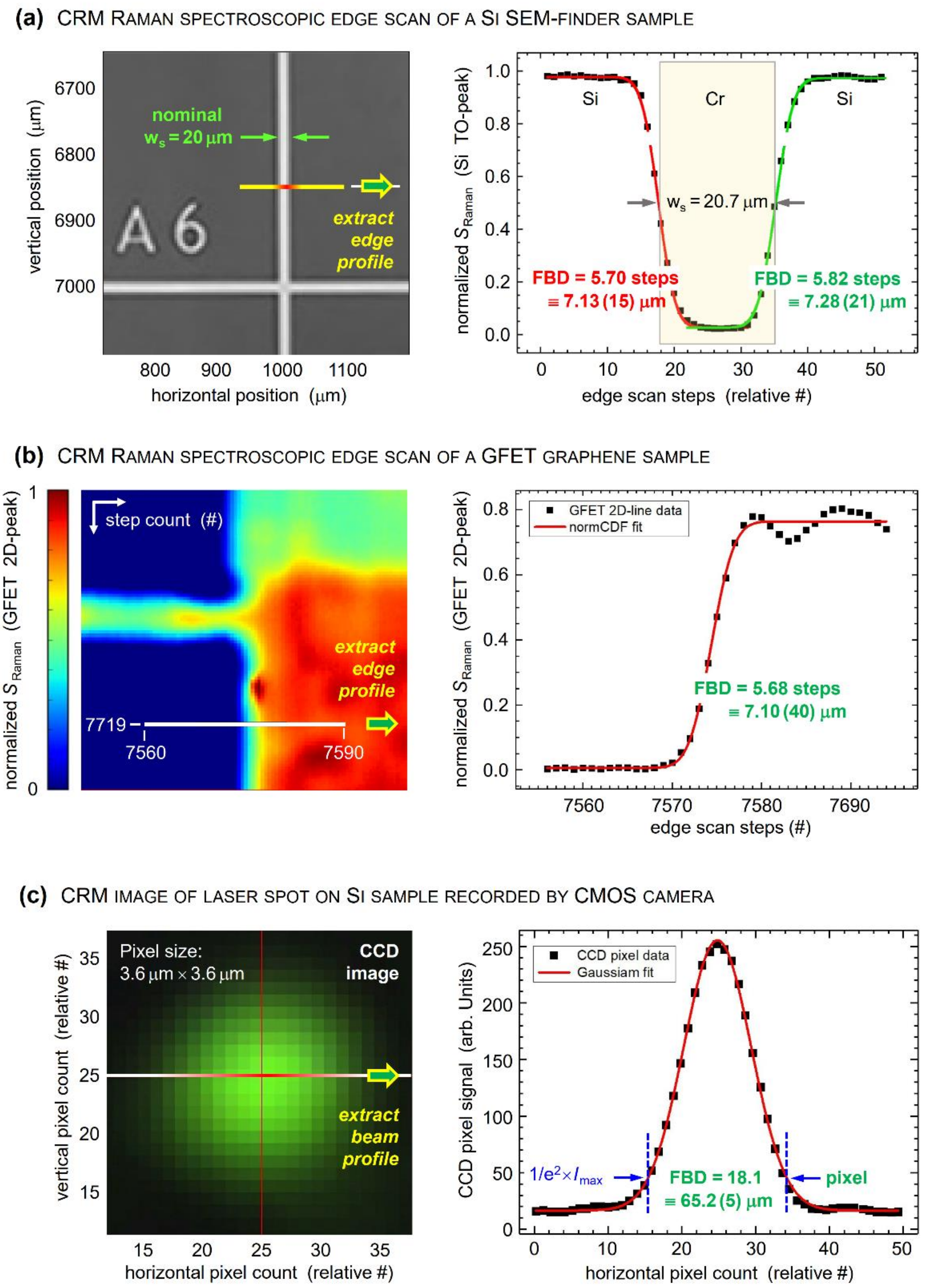

Figure S2. Determination of the laser focal beam diameter (= FBD) on the target surface, through the ×10 microscope objective. (a) Line-scan for the focussed laser beam across a chromium grid line of a silicon SEM-finder grid substrate (EM-Tec FG1, *Micro to Nano*), using the edge-scan method [RS6]; the scan data are for the 2TO spectral line of silicon. (b) Line-scan for the focussed laser beam on a GFET graphene sample (GFET-S10, *Graphenea*), using the edge-scan method; the scan data are for the 2D spectral line of graphene. (c) Laser beam spot imaged onto the CRM CMOS-camera, and the determination of its beam waist (BW) from a Gaussian fit. For details, see text.

Note also that in our knife-edge scan $P_N(x)$ does not represent the actual laser power but rather the Raman response signal resulting from the applied laser power

In the data plot of Figure S2(a) fits of the leading (red line) and trailing (green line) edges to equation (6) are included; these fits replicate the experimental data points rather well. From these data fits the laser focal beam diameter (FBD) can be determined; here we define as beam diameter as the $1/e^2$ point in the Gaussian TEM_{00} profile function.

The average over the eight leading/trailing edges of the four grid lines contained in the scan yielded FBD = 7.23(13) μm on target (the statistical error value is linked to the last numerical digit of the average value). The following additional observations can be made, based on the results from this finder grid scan.

First, the spacing between Cr-grid lines was determined experimentally as 800.0 ± 0.2 steps, which is equivalent to 1000.0 ± 0.25 μm; this replicates the nominal grid line spacing within the mechanical accuracy of the stepper driver of the translation stage.

Second, the experimental width of the Cr-grid lines, as determined from the half-intensity points of the knife-edge scan(s), is found to be $w_{GL} = 20.5 \pm 0.3$ μm. This value is slightly larger (about 2-3 %) than the nominal value stated in the data sheet, which is most likely associated with the uncertainty in the analysis methodology based on (eq.3).

Finally, we would like to note that many other structured reference materials are suitable for application of the knife-edge method in confocal (Raman) microscopy. For example, Trägårdh and co-workers [35] used a simple transmission electron microscopy (TEM) grid specimen – with 40×40 μm^2 grid squares – mounted on a glass microscope slide, for a variety of different microscope objectives. In the case that very high magnification is utilized (such as e.g. a 100× objective), substrates with ultra-precise edge structures on a small scale are required. In this context, Itoh and Hanari [36] used a tungsten-dot array (on silicon substrate) certified reference sample in their confocal Raman microscope to determine the lateral resolution of their system, and associated with that the laser focal beam diameter on target.

In order to establish that in principle any sample, which exhibits a sharp transition from one material to another, is suitable to determine the laser beam waist by the knife-edge method (in case no reference such as a finder grid sample is available) we performed a line scan across the graphene area of a GFET device of the aforementioned graphene sample substrate.

We like to note here that this type of approach is not entirely new: determining the laser spot size using a graphene sheet, mounted on a Si-substrate, has been reported e.g. by Sacco *et al* [37]. It has to be noted as well that the edge information gained from such a graphene-on-silicon sample is likely less precise: the difference between Si-signal / no-Si-signal when scanning across any of the Cr-metal grid lines does not suffer from interference as the graphene sample does. For the latter always contributions from the Si-substrate are carried along, which makes the signal contrast during the scan much worse.

Line scan data for our silicon-to-graphene edge were extracted from a partial raster scan of a GFET sample (the one shown in the left data panel of Figure S2(b). One particular line scan is indicated by the white line (with related pixel positions); the associated knife-edge evaluation data are displayed in the right data panel. The evaluation of this edge scan yields FBD = 7.10 (40) μm on target. As before, the cited fit error relates to the last numerical digit of the average value. Two observations can be made.

First, from the displayed raster scan map one it is clear that the edge is not exactly straight but “rugged” and that, on first impression, the steepness of the edge data seem to vary in the “vertical” scan coordinate. Therefore, in principle one would need to take the average over a number of line scans to obtain a better feeling for the quality and reliability of the result. Most likely the observed x-y structural shape in the edge is dictated by how the graphene flake has been generated and how it was transferred and affixed to the GFET substrate.

Second, the knife-edge data shown in Figure S2(b) exhibit an “oscillatory” behavior, which in turn results in a less precise fit value according to equation (6), in comparison to the SEM finder grid data. Such an undulation in the Raman data has also been observed by Sacco *et al* [37]). Most likely this is associated with the stress in the graphene flake toward its edges, specifically when mounted on a substrate with different lattice constant.

Overall, the results from the two measurement campaigns – the Cr-metal lines on Si-mono-crystal and the graphene square of the GFET device – yield, within their uncertainty limits, a consistent value for the laser FBD on target.

Note that the laser FBD on target is always monitored by imaging it on the CRM's CMOS-camera; by minimizing the size of this image it is ascertained that the sample surface is within the focus of the laser beam.

In principle, one should be able to deduce the laser FBD on target from the camera image (see Figure S2(c)). By applying Gaussian beam propagation principles one may back-reconstruct the focal laser beam waist on target, $w_{0,\text{@sample}}$, from the image with waist, $w_{0,\text{@CCD}}$:

$$w_{0,\text{@CCD}} = (\lambda \cdot f_{\text{acr}})/(\pi \cdot w_{\text{f,acr}}) \Leftrightarrow (\lambda \cdot f_{\text{obj}})/(\pi \cdot w_{\text{f,obj}}) = w_{0,\text{@sample}} \; . \qquad \text{(eq.4)}$$

Here the index "acr" stands for the achromatic lens used for imaging the light reflected / emitted from the sample onto the CMOS detector; the index "obj" is associated with microscope objective; w_0 and w_f stand for the focused and collimated beam waist parameters, respectively; and λ is the laser wavelength.

However, a direct back-reconstruction relation between the two parts of (eq.4) might only be established if exact collimated beam propagation within the CRM prevails, i.e. $w_{\text{f,acr}} \equiv w_{\text{f,obj}}$. This is not necessarily the case since the beam from the entrance fiber collimator is slightly divergent.

Furthermore, (eq.4) is derived for the propagation through thin lenses; neither the objective nor the imaging achromatic lens can be treated as thin optical elements. Therefore, it is not surprising that, applying (eq.4) as a back-reconstruction tool to derive the laser FBD on target from the camera image does not yield a value which replicates the one obtained via the (direct) knife-edge method. A full ray-tracing treatment through thick optical elements would be required for matching the results from the two methodologies. Nevertheless, for our CRM configuration the back-projected laser FBD on target yielded a value, which differs by about 12 %; this still can be judged as being a reasonable approximation.

Finally, it is worth noting that the laser spot size on target achieved here does not constitute the ultimate (diffraction) limit. Because for simplicity of alignment the laser radiation is fed into the CRM system using a single-mode fiber (*Thorlabs* P1-460B-FC-2) coupled to a fixed-focus fiber-collimator (*Thorlabs* F260FC-A). The associated laser beam waist of ~2.8 mm is substantially less than the diameter of the objective's rear aperture of ~9 mm. As a consequence, the experimental laser beam spot size is quite a bit larger than the theoretical limit in the case that the full numerical aperture of the objective, NA_{obj}, be used for laser illumination (according to the common approximation $w_{0,\text{@sample}} \cong \lambda/(\pi \cdot \text{NA}_{\text{obj}})$ the laser FBD on target would be of the order 2.5 μm).

References S.A.3:

[25] Khosrofian, J.M.; Garetz, B.A. Measurement of a Gaussian laser beam diameter through the direct inversion of knife-edge data. *Appl. Opt*. **1983**, 22, 3406-3410. https://doi.org/10.1364/AO.22.003406

[26] de Araújo, M.A.C.; Silva, R.; de Lima, E.; Pereira, D.P.; de Oliveira, P.C. Measurement of Gaussian laser beam radius using the knife-edge technique: improvement on data analysis. *Appl. Opt*. **2009**, 48, 393-396. https://doi.org/10.1364/AO.48.000393

[35] Trägårdh, J.; MacRae, K.; Travis, C.; Amor, R.; Norris, G.; Wilson, S.H.; Oppo, G.L.; McConnell, G. A simple but precise method for quantitative measurement of the quality of the laser focus in a scanning optical microscope. *J. Microscopy* **2015**, 259, 66-73.[https://doi.org/10.1111/jmi.12249

[36] Itoh, N.; Hanari, N. Reliable evaluation of the lateral resolution of a confocal Raman microscope by using the tungsten-dot array certified reference material. *Anal. Sci*. **2020**, 36, 1009-1013. https://doi.org/10.2116/analsci.20P046

[37] Sacco, A.; Portesi, C.; Giovannozzi, A.M.; Rossi, A.M. Graphene edge method for three-dimensional probing of Raman microscopes focal volumes. *J. Raman Spectrosc*. **2021**, 52, 1671-1684. https://doi.org/10.1002/jrs.6187

S.A.4 – Automatic control for keeping samples in the laser focus

For the generation of planar (xy-direction in our geometry frame) images of a sample, during the scan the sample surface needs to be stay within the laser focus (z-direction). Due to unavoidable offset and rotations in the assembled xyz-motion-stage and uneven mounting in the sample holder, the sample and the laser

focus planes are not necessarily parallel to each other on a sub-degree level. Thus, the sample surface would likely move out of focus during a full planar scan.

This is a general problem in whole-slide (laser) microscopy, in which topographic variability may severely affect in-focus imaging. In a recent review article Bian *et al* [38] compare so-called (traditional) focus map approaches with various real-time autofocusing methodologies. However, it needs to be noted that these are most suitable for situations, in which direct area-imaging is utilized – not pixel-by-pixel scanning – to keep a particular topographic segment in focus.

In the raster-scan Raman imaging described here, which at present only utilizes a low-magnification 10× objective, normally topographical variations are well within the standard focal depth a few tens of μm. However, as mentioned earlier, the sample surface may not be absolutely in-plane for lateral xy-scans, i.e. the sample might be mounted with a tilt. Therefore, the surface may severely run out of focus during a full-area raster scan; for a compensated scan-procedure knowledge of the particular sample tilt is required.

In order to correct for the tilt of the sample, the tilt angles $\theta_{x,y}$ need to be measured *in-situ*. Using the CMOS camera three different points P_1, P_2, P_3 on the sample surface are selected such that all points are in the focus of the laser beam. From these three points two vectors $r_{x,y}$ on the sample surface are constructed. Finally, the vector dot-product of $r_{x,y}$ with the normal vector $n_z = (0,0,1)$ is used to calculate the angles

$$\theta_{x,y} = \cos^{-1}\left(\frac{\vec{n}_z \cdot \vec{r}_{x,y}}{|\vec{n}_z| \cdot |\vec{r}_{x,y}|}\right) . \qquad \text{(eq.5)}$$

The coordinate system of the xyz-motion-stage is then "rotated" using basic rotation matrices R_x and R_y, according to

$$P_i = R_y(\theta_y) \cdot R_x(\theta_x) \cdot P_i' \ , \qquad \text{(eq.6)}$$

where P' denotes the original tilted system and P is the corrected system. This procedure is implemented in our bespoke *LabView* software package in such a way that the user enters coordinates only in the corrected coordinate system, which then are back-transformed and transmitted to the xyz-motion-stage for movement.

With the corrected coordinate system, the z-coordinate remains constant during the planar scan and the sample is kept in the laser focus. This procedure is applied first, whenever a new sample is installed in the sample holder.

References S.A.4:

[38] Bian, Z.; Guo, C.; Jiang, S.; Zhu, J.; Wang, R.; Song, P.; Zhang, Z.; Hoshino, K.; Zheng, G. Autofocusing technologies for whole slide imaging and automated microscopy. *J. Biophotonics* **2020**, 13, e202000227. https://doi.org/10.1002/jbio.202000227

S.A.5 – Axial scanning

While every effort had been made – during the initial system alignment – to position the wide-field camera chip and the confocal pinhole to be simultaneously in their respective focal planes, for the Raman measurements the two signal maxima might not necessarily exactly match for thin-film layered structures, which in addition might be (partially) transparent, resulting in camera images from different surfaces. Therefore, axial profile scans were carried out, moving the sample surface through the laser focus, roughly from 200 μm above to 200 μm below the laser focus, while recording Raman spectra for each axial position.

For the determination / demonstration of the depth (axial) resolution, two different graphene samples from *Graphenea* were scanned along the z-axis, namely (a) a graphene-on-glass substrate; and (b) a GFET-S10 device. From the stack of spectra, relevant interval slices of ~100 cm^{-1} were selected (at the spectral positions of the SiO_2 TO-line, and the graphene 2D- and D-lines, respectively).

The Raman spectral intensities within the slice intervals were integrated, and subsequently were normalized to the observed maximum signal value. In Figure S2 these data were then plotted as a function of the axial distance z_R from the surface, with the graphene monolayer at z_R=0. As can be seen in the figure, the response functions for all evaluated Raman lines mimic very much the passage of the graphene layer

through the focal position of the propagating Gaussian beam, with the common definition of the focal depth L (Rayleigh length):

$$L = 2z_R = 2\pi w_0^2/\lambda_{\text{las}} \;, \qquad \text{(eq.7)}$$

here z_R is the axial position at which the beam area has doubled, i.e. to $w_z = \sqrt{2} \cdot w_0$, and λ_{las} is the laser wavelength. By and large, the Raman signal response follows the expected integral laser intensity within the confocally-imaged interaction volume. For this recall here that, the axial intensity distribution either side of the laser focus can roughly be approximated by a Lorentz profile function (see e.g. [39]).

Note that the profiles displayed in Figure S3 do not seem fully symmetric around the focal position, z_0. This can be associated with imperfections in the confocal imaging through a pinhole, including effects such as spherical aberration and depth scale compression [39]. However, this asymmetry is only minimal at the low magnification of 10× utilized in these measurements.

From the Lorentzian fit to the data traces in Figure S3 one can deduce the experimental focal depth $L = 2z_R$ of the current CRM configuration as $L_{\text{exp}} = 44 \pm 5$ µm (for clarity only shown in part (b) of the figure). This is reasonably consistent with the theoretical value one arrives at when using equation (3), with the value for w_0 as determined from the laser spot size on target (see Section A.3 above), namely $L_{\text{theor}} = 38 \pm 2$ µm.

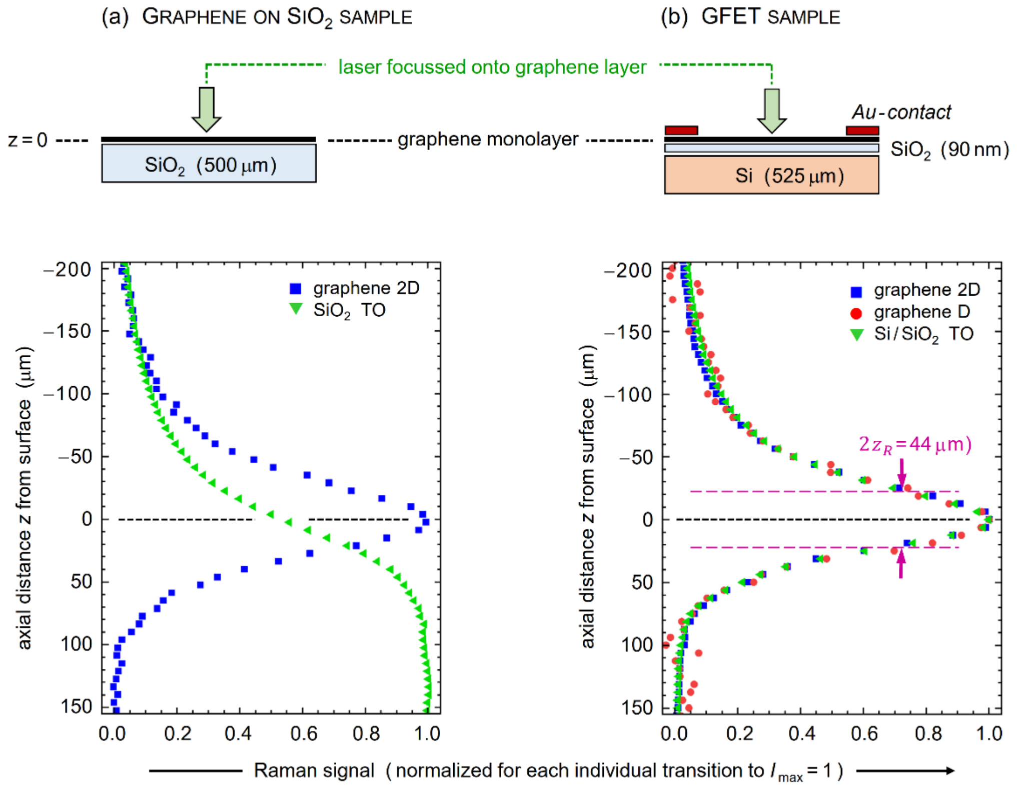

Figure S3. Axial (depth) profiling for single-layer graphene: (a) graphene on a glass (fused silica) substrate and (b) graphene on a SiO_2/Si substrate, forming part of a GFET device structure (GFET-S10, *Graphenea*). The scans for both samples are for the laser focus range from −200 µm above to +200 µm below the graphene layer, in step increments of ΔS = 5 (≅ 6.5 µm). The Raman signal responses for the Si(2TO) and graphene(D,2D) lines are shown.

In principle, one also might evaluate the depth resolution in Raman axial scans (see e.g. [40]); however, this has not been within the remit of this project – only monolayer graphene samples were to be probed. In this context it should be noted that, for layers of a given thickness the observed axial profile would roughly replicate a Lorentzian point spread function convoluted with a Top-Hat function whose width is equivalent to the layer thickness. Evidence of this can be seen in Figure S3(a) for the TO-line Raman response trace from the (transparent) SiO_2 substrate (the falling edge at the lower substrate boundary is outside the figure scale). Of course, for proper µm-scale depth profiling high-magnification objectives need to be used.

References S.A.5

[39] Everall, N.J. Confocal Raman microscopy: performance, pitfalls, and best practice. *Appl. Spectrosc*. **2009**, 63, 245A-262A. https://doi.org/10.1366/000370209789379196

[40] Everall, N.J. Confocal Raman microscopy: common errors and artefacts. *Analyst* **2010**, 135, 2512-2522. https://doi.org/10.1039/c0an00371a

S.A.6 – The use of different pin holes and objectives

For the ease of modelling the image formation through a confocal microscope one may approximate both the objective and the focusing achromatic lens as radially-symmetric, aberration-free ideal optical lenses with circular aperture. Regardless, the image formed of a (monochromatic) point source – mathematically described by a point spread function (PSF) – is in any case limited by diffraction at the edges of the objective / lens aperture(s). This lateral intensity profile, formed in the plane of the confocal pinhole, can in the ideal case be described by the Airy function in the radial direction (see e.g. [41]). In the related Airy-pattern the central part between the first minima of the intensity distribution is normally cited as the Airy-disc; it contains the bulk fraction of the total light intensity, and the Airy disc diameter is often used as a measurement unit for pinholes and spatial resolution, with Airy-disc diameter ≡ 1 Airy unit [AU].

It should be noted that this simplified approach needs to be modified if one wishes to determine an exact PSF through an actual confocal microscope (see e.g. [42]).

The pinhole is one key element affecting the performance of a confocal (Raman) microscope; therefore, its dimension (and position in the beam path) need to be considered carefully. In general, as a good compromise, the pinhole diameter is chosen to equal that of the Airy-disk diameter in confocal imaging. Detailed considerations of the pinhole diameter and its effect on resolution may be found e.g. in references [27-29].

The size of the Airy-disk ($\emptyset_{\text{Airy disk}}$), and therefore the confocal pinhole ($\emptyset_{\text{confocal pinhole}}$), depends on the numerical aperture of the objective lens (NA_{obj}); the wavelength (λ) of the imaged light; and any magnification (M_{CRM}) up to the pinhole:

$$\emptyset_{\text{confocal pinhole}} \equiv \emptyset_{\text{Airy disk}} \approx 2.44 \times \left(\frac{\lambda}{2 \cdot NA_{obj}}\right) \times M_{CRM} \; . \qquad \text{(eq.8)}$$

Note therefore that, as a consequence of (eq.6), a different pinhole size is required if the objective lens is changed to one with different NA_{obj}. However, note as well that, this equation is valid under the proviso that the full aperture of the objective is exploited, as is the case in standard confocal microscopes and confocal laser-induced fluorescence microscopes. Here we are dealing with a confocal Raman configuration for which particular conditions apply, as will be commented on below.

For the majority of the work discussed in this publication we used a 10× infinity-corrected objective with $NA_{obj} = 0.25$; the magnification of the CRM of about 8.3 (approximated from the focal lengths of the objective ($f_{obj} = 18$ mm) and the imaging achromatic lens ($f_{acr} = 150$ mm)). The wavelength range covered extends from the laser excitation ($\lambda_{las} = 532$ nm) to just beyond the 2D-peak of graphene ($\lambda_{Raman} = 632$ nm).

Using (eq.8) this would suggest a theoretical confocal pinhole diameter of $\emptyset_{\text{confocal pinhole}} \sim 25\ \mu m$. However, for our CRM setup this value is erroneous since – as mentioned before – not the full objective aperture is utilized. This is because (i) the laser illumination has a much narrower beam waist; and (ii) the Raman light maintains much of the directionality of the exciting light (in contrast to the scenario of laser-induced fluorescence). As a compromise, we therefore based our estimate of the confocal pinhole diameter on the experimental FBD of the camera image, formed from the laser spot on target. This image should be roughly equivalent in its FBD at the plane of the pinhole. Correlating the Airy-function to a Gaussian function (see e.g. [43]) – which is a common approach for ease of calculation – one finds that $\emptyset_{\text{Airy disk}} \approx 1.25 \times FBD_{\text{CCD Gaussian}}$. With the value derived in Figure S2(c) $FBD_{\text{CCD Gaussian}} = 65\ \mu m$ one therefore obtains for the optimal pinhole diameter the value of $\emptyset_{\text{confocal pinhole}} \approx 82\ \mu m$.

In our studies we have used a range of pinholes, namely standard mounted pinholes (*Thorlabs*) of 100 μm, 75 μm, 50 μm and 25 μm diameter; for the current CRM setup that means sizes equivalent to ~1.25 AU, ~1 AU, ~0.6 AU and ~0.3 AU, respectively. When measuring the intensity profiles in lateral and axial direction with these four pinholes, by and large the results mimic those expected from theory (see e.g. the confocal

performance plots in references [27,28]). Note that most of the data discussed in this main text of our publication were recorded using the pinhole of 100 μm diameter, which maximized the Raman light signal without sacrificing confocality.

References S.A.6

[27] Wilson, T. Resolution and optical sectioning in the confocal microscope. *J. Microscopy* **2011**, 244, Pt 2, 113-121. https://doi.org/10.1111/j.1365-2818.2011.03549.x

[28] Borlinghaus, R.T. Super-resolution: on a heuristic point of view about the resolution of a light microscope. Technological Readings, Leica Microsystems CMS GmbH, Mannheim Germany, December **2014**. Online access: https://downloads.leica-microsystems.com/Leica%20TCS%20SP8%20MP/Dedicated%20Articles/Super-Resolution_Technological%20Readings_Dec2014.pdf Last accessed: Dec 10, 2022.

[29] Borlinghaus, R.T. Pinhole effect in confocal microscopes. Science Lab - the knowledge portal of Leica Microsystems CMS GmbH, Mannheim, Germany, April 26, **2017**. Online acces: https://www.leica-microsystems.com/science-lab/pinhole-effect-in-confocal-microscopes/ Last accessed: Dec 10, 2022.

[41] Vallee, O.; Soares, M.; Soares, M. Airy functions and applications to physics, 2nd ed. Imperial College Press, London, UK, **2010**.

[42] Braat, J.J.M.; Dirksen, P.; van Haver, S.; Janssen, A.J.E.M. Extended Nijboer-Zernike (ENZ) analysis and aberration retrieval (last update: 25 February, 2022). Online access: [https://nijboerzernike.nl/ Last accessed: Dec 10, 2022.

[43] Garcia-Lechuga, M.; Grojo, D. Simple and robust method for determination of laser fluence thresholds for material modifications: an extension of Liu's approach to imperfect beams. *Open Res. Europe* **2021**, 1, 7. https://doi.org/10.12688/openreseurope.13073.2

S.B. Measurements of selected samples

In order to ascertain that apparent spectro-spatial features could be associated with real spatial differences in the sample, we performed a series of test measurements in which we compared the spectrochemical raster maps with images of the same area obtained using different imaging techniques.

In particular, we recorded (i) SEM images of small surface areas of GFET devices; and (ii) wide-field light images of a large surface area of a graphene-on-glass sample. These were then overlaid with the Raman images acquired with our CRM instrument. Two examples are discussed in Sections B.1 and B.2 below.

S.B.1 – Raster scans of graphene sheets and GFET sensing devices

In order to demonstrate the capabilities of our CRM system for spatially-resolved spectrochemical analysis we executed a series of raster scans of graphene samples, predominantly GFET devices (*Graphenea*) with different sizes of the graphene area.

As pointed in the main text, in principle the full area of the GFET multi-device substrate of 10×10 mm^2 could be scanned, with μm-resolution. However, in the proof-of-principle scans discussed here, in general raster maps were limited to areas of the order 50×50 raster points, in order to keep the total acquisition time to a reasonable duration. Note that two back-to-back acquisitions were taken at each raster point location, of the order of 10-20 s duration each (this dual-spectra acquisition is required to apply the LARAsoft-internal cosmic-ray removal procedure [16]).

The lateral step increments were adjusted to match the properties of the laser focal beam diameter (here FBD ~ 5.7 steps, equivalent to ~7 μm). For quick survey scans step increments equal or larger than the FBD were utilized (ΔS = 5-10), while for the purpose of revealing finer details in the sample surface structure step increments of ΔS = 1-2 were usually selected. Note that a full-step increment ΔS = 1 corresponds to a spatial displacement of 1.25 μm.

Because of the inherent backlash in the motorized translation stages, the raster scans were normally executed unidirectional to avoid line-by-line distortions in the Raman images. Note that from forward / backward scans across four sequential Cr-rid lines of the SEM finder grid sample we determined this backlash to be ~1.8 full-steps. Thus, the raster maps constitute consecutive line-scans in (horizontal) x-direction, returning to the x-reference location after completion; for the subsequent x-direction scan the sample position was incremented in (vertical) y-direction by the chosen ΔS.

Here we also would like to note that for the xyz-stage implementation in our UAM system – using the open-loop piezo-inertia translators PD1/M (*Thorlabs*) – a software solution needed to be implemented to achieve the required positional repeatability, because of the different step size of the piezo-inertia actuator when reversing the direction (see e.g. [44]). In order to avoid this type of complex software code and calibration procedure, replacing the open-loop devices (PD1/M) with closed-loop units (PD1X/M with incorporated optical encoder, *Thorlabs*) circumvents this problem albeit at increased cost.

After completion of the full raster-scan, the hyperspectral data cube was analyzed according to the procedure described in Section 4.1 of the main text. Note that for simplicity the spectrochemical maps shown here only represent single-peak slice evaluations rather than full hyperspectral analysis, as we stated before. In difference to the raw-spectra shown in Figure 3 in Section 4.1 of the main text, for the data discussed here the spectra were corrected for fluorescence and stray light background prior to spectral slicing, using the LARAsoft internal SCARF routine (see ref. [16]).

Afterwards, the treated data cube was evaluated for selected spectral slices, namely at the locations of the graphene peaks D, G and 2D (at ~1350 cm^{-1}, ~1600 cm^{-1} and ~2700 cm^{-1}, respectively); and for reference at the Si / SiO_2 peaks TO and 2TO (at 520 cm^{-1} and 970 cm^{-1}, respectively).

As an example for spectrochemical mapping, the evaluation of a raster-scan of a GFET device (200×200 μm^2, *Graphenea*) is shown in Figure S4. The sequence displays the same area segment of the device – indicated in panel (a) of the figure – with a series of different step resolutions, namely $\Delta S = 7$, $\Delta S = 4$ and $\Delta S = 2$ steps (corresponding to spatial increments of 8.75 μm, 5.0 μm and 2.5 μm, respectively). The raster maps shown here are for spectral slices for the three main graphene peaks. The following observations can be made:

First, all spectral raster maps clearly reveal the “edges” between the graphene chip and the Si / SiO_2 substrate (or metal contacts), regardless of spatial step-increment – different in data panels (b) to (d), equal in data panels (d) to (f)).

Second, the three data panels with different spatial step-increment – for the 2D peak of graphene – all exhibit certain “inhomogeneities” across the displayed area. However, observed “features” do not seem to correlate well with each other in the three scans, although some semblance may be interpreted. This notional mismatch is likely associated with the fact that the three raster-scans were acquired during different days. Although every effort had been made to keep operational conditions as constant as possible (in particular the laser power was adjusted to be equal within 1-2%), effects like temperature drift or variation in fluorescence / scattered light background were difficult to ascertain. Certainly, further repeat measurements would be required to provide statistical information for each individual raster pixel.

Third, the spectral image maps shown in panels (d) to (f) for the different graphene peaks stem from the same raster-scan and thus are directly correlated – in contrast to the sequence shown in panels (b) to (d). With the spatial step increment $\Delta S = 2$ steps ≡ 2.5 μm (substantially smaller than the laser spot with FSD ≅ 7.1 μm) some “structural features” can be identified as being common to all three spectral images, although they become increasingly blurred in the sequence (d) to (f) due to the decreasing signal intensities – notably $I_{\text{peak-2D}} > I_{\text{peak-G}} \gg I_{\text{peak-D}}$ –, and thus a noisier signal.

References S.B.1

[16] James, T.M.; Schlösser, M.; Lewis, R.J.; Fischer, S.; Bornschein, B.; Telle, H.H. Automated quantitative spectroscopic analysis combining background subtraction, cosmic ray removal, and peak fitting. *Appl. Spectrosc*. **2013**, 67, 949–959. https://doi.org/10.1366/12-06766

[44] Hunstig, M. Piezoelectric Inertia Motors—A critical review of history, concepts, design, applications, and perspectives. *Actuators* **2017**, *6*, 7. https://doi.org/10.3390/act6010007

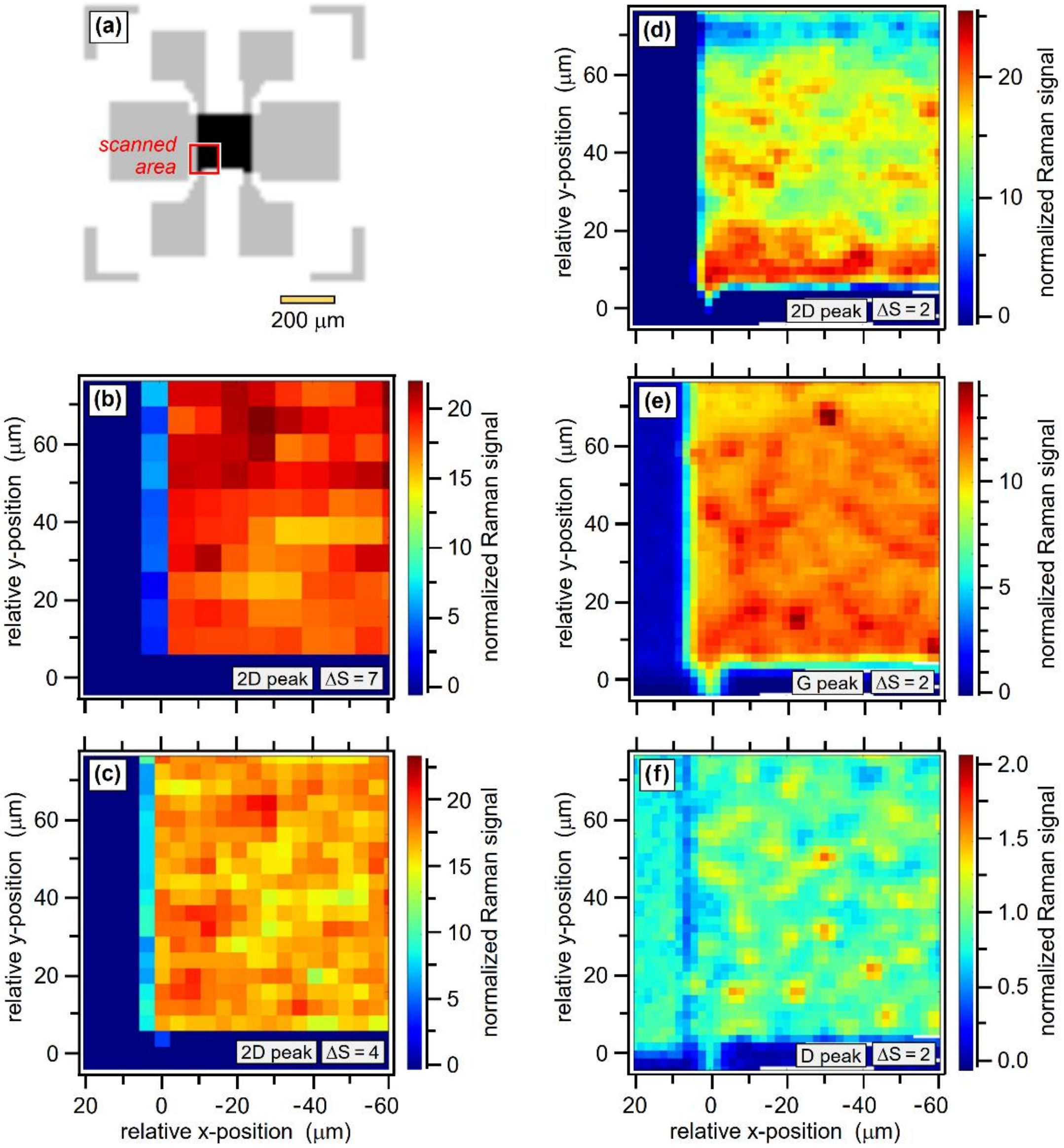

Figure S4. Raman image maps of a GFET device (200×200 μm^2, *Graphenea*). (a) Schematic GFET structure; the area scanned by CRM – about 80×80 μm^2 – is indicated by the red square; the spatial reference point for the scans is the left-most contact. (b) Map of the 2D-peak signal, scanned with a step increment of ΔS=7. (c) Map of the 2D-peak signal, scanned with a step increment of ΔS=4. (d) – (f) Maps of the 2D-, G- and D-peak signal, respectively, scanned with a step increment of ΔS=2. Note that 1 step-increment (ΔS=1) corresponds to a spatial displacement of ~1.25 μm. For further details, see text.

S.B.2 – Comparison of Raman raster maps vs SEM images: GFET device sample

The SEM image of the GFET device, shown in Figure S5, reveals two structural features. Namely, these are (i) graphene-flake boundaries, evident from the faint “black” lines in the image; and (ii) a series of local, circular-shaped features – most likely from deposits during chip manufacture, or adhesion of small dust particles during sample handing in ambient air. Note that both features are also present in the SEM images of GFET devices included in the *Graphenea* GFET-S10 data sheet and on their webpage [45].

The overlay between this SEM image and the Raman raster map for the D-peak of graphene is shown in Figure S5(b); note that this peak was selected for display since it is the most sensitive for any deviation from the pure-graphene monolayer signal. The cross (in red) in both images is the spatial reference location used, i.e. the corner of the graphene chip.

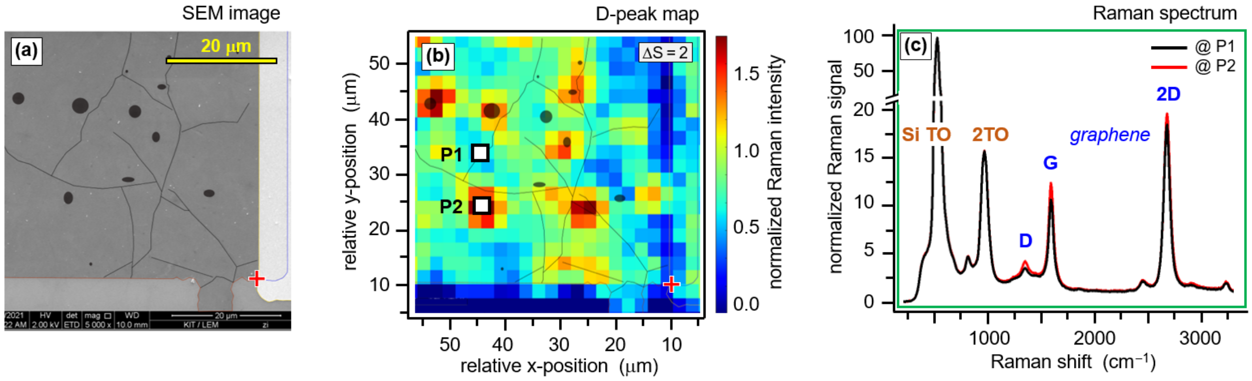

Figure S5. Comparison of confocal Raman spectral maps with SEM image. (a) SEM image of a 200×200 μm^2 GFET sample, contrast-enhanced to emphasize graphene flake boundaries and defect areas. (b) Raman scan map for the graphene D-peak of the same sample area shown in (a). (c) Raman spectra from the locations marked with the (white) squares in the Raman raster map (b). Note that the spatial reference points between the images / Raman maps are marked with (red) crosses.

It is clear that the fine border lines between graphene-flakes – where some deviation in the lateral graphene response signal would be expected – are not really discernible in the Raman map, This is not surprising because the spatial resolution with the 10× objective is insufficient to do so. However, most of the circular (deposit) features exhibit reasonable correlation between the two images albeit somewhat blurred due to the inherent limited lateral resolution. For comparison of on-deposit / off-deposit Raman signals the full (raw) spectra at the related spatial positions are shown in Figure S5(c). From these, subtle differences in the spectral fingerprints are evident, with the most prominent change in Raman response in the D-peak.

Despite the evident correlation it is clear that the single-slice evaluation of the spectra is insufficient for a meaningful interpretation of the observed features but that full hyperspectral analysis would be required, as well – most likely – increased spatial resolution.

References S.B.2

[45] GFET-S10 for Sensing applications. Graphenea, San Sebastian, Spain. Online access: https://www.graphenea.com/products/gfet-s10-for-sensing-applications-10-mm-x-10-mm . Last accessed: Dec 10, 2022.

S.B.3 – Comparison of Raman raster map vs wide-field image: Graphene-on-glass sample

As was pointed out in the introduction to our publication, one of the initial purposes of setting up this custom-built CRM was to be able to analyze graphene samples before, during and after exposure to tritium or larger tritium-substituted molecules, to monitor chemical bonding changes in graphene and to ascertain tritium loading. In order to demonstrate this desired application, without potential radioactive contamination of the CRM prior to the actual tritium campaigns, a preliminary "quick-and-dirty" study was undertaken to load graphene with (atomic) hydrogen. In this context, we exposed a graphene-on-glass sample [46] to a solution of H_2SO_4, one of the potential electrochemical methodologies discussed in the literature to achieve hydrogen bonding, i.e. H-graphene (see e.g. references [47]).

For this, the graphene sample was dipped into a dilute sulfuric-acid solution for a few minutes. In addition to the Raman raster scanning attempts were made to measure the sample conductivity, to confirm hydrogenation of the graphene layer (see e.g. references [48]); note that structural / chemical changes in graphene are known to lead to altered conductivity, see e.g. references [47,49]).

In Figure S6(a) a white-light wide-field image of the graphene-on-glass sample is shown, after it was partially exposed to the sulfuric-acid solution. In contrast to the rather homogeneous white-light image of the pristine sample (not shown here), the exposed sample clearly shows significant damage to the graphene layer, caused in part by its "rough" handling (dipping by hand, clamping of electrical clip-connectors). Ruptures in the surface as well as ruggedized edges are visible; of cause, any chemical changes are not revealed in this white-light image.

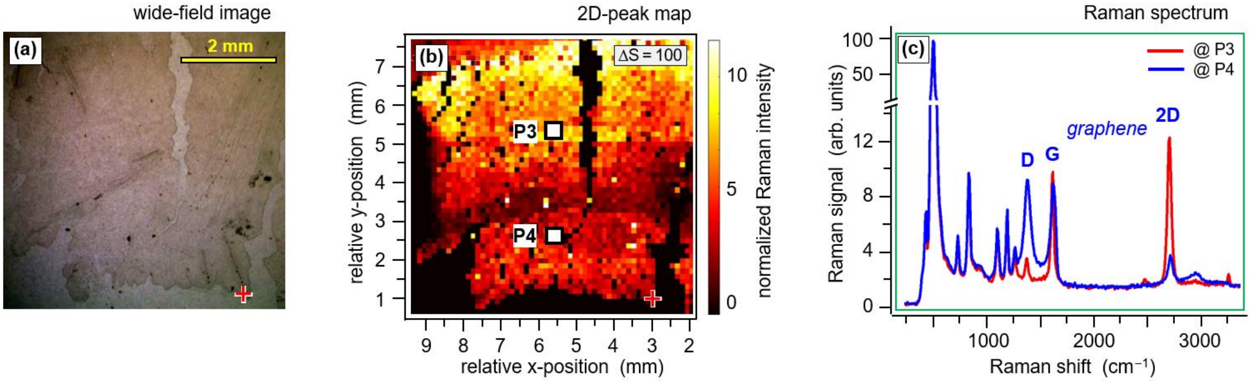

Figure S6. Comparison of confocal Raman spectral maps with wide-field CCD image. (a) Wide-field image of graphene on fused silica (note that the sample shown here was exposed to H_2SO_4 for hydrogenation). (b) Raman scan map for the graphene 2D-peak of the same sample area shown in (a). (c) Raman spectra from the locations marked with the (white) squares in the Raman raster map (e); spectra recorded with increased spectral resolution. Note that the spatial reference points between the images / Raman maps are marked with (red) crosses.

In order to confirm that hydrogenation (or other chemical changes) of graphene was achieved a Raman raster scan was performed, covering the complete area shown in the white-light image, i.e. an area of roughly 7.5×7.5 mm^2 (out of the total graphene sheet of size 10×10 mm^2). Clearly, a scan with the same resolution as shown for the GFET sample would have meant to record spectra for of the order of 10^7 raster points, thus equating to an excessively long measurement time. Therefore, the sample was advanced in increments of $\Delta S = 100$ steps, or 125 μm; consequently, maps of ~60×60 raster points ensue. It should be noted that, this procedure does not generate gapless maps of the sample but only probes it at sparse spatial locations.

As an example, the spectrochemical map for the 2D-peak of graphene is depicted in Figure S6(b). Note that, as before, the (red) cross indicates the reference points for the two imaging methods. Clearly, the ruptures and edges are replicated well in the Raman map, associated with a zero-2D-peak signal (black in the amplitude scale of the map). Finer details visible in the white-light image cannot always be directly correlated to the Raman map; this is a consequence of the widely spaced raster points across the sample surface. However, a few things are recognizable.

Despite the wide-space raster scan, the Raman signal is not homogeneous across the full sample area. In particular, irregular “spikes” are visible. With the help of the Raman spectra in Figure S6(c) (in the display not corrected for fluorescence background), associated with the raster points P3 and P4, can be used for chemical characterization. Data points with high 2D-peak amplitude still resemble graphene, although one would need to do multi-spectral or hyperspectral analysis to extract subtle details. Data points with low 2D-peak amplitude indicate chemical bond modification, most likely hydrogenation; for a comparison of Raman spectra for graphene vs hydrogenated graphene see e.g. references [47]. Note however, that similar spectral changes are also observed for oxidized graphene (see e.g. the spectra in reference [50]). Thus, as stated earlier, single-peak analysis is insufficient to identify chemical species unequivocally, but only that a chemical bond change with respect to a pristine graphene sample may be recognized.

In addition to the observed signal spikes, a more-or-less gradual trend from modified to unmodified graphene is evident toward the top of the Raman map image. This can be associated to the depth to which the sample was dipped into the sulfuric acid. The transition is not abrupt but gradual because of the upward creep of the acid solution associated with surface tension, and thus gradual reduction in hydrogenating agent. Regardless, local inhomogeneities are always expected, even if the exposure to reagents is much more controlled, and likely depends on the duration of the exposure as well (see for example the gradual increase of hydrogenation with time in Figure 4.6 of reference [51]).

Overall, the CRM imaging results discussed here in Sections B.2 and B.3 show two things. First, in general, spatial features in the Raman maps can be linked to the features detected by alternative analysis methods at the same position. Second, differences of spectral response are observed for said features. However, single-peak spectral slice evaluation is often insufficient to determine the actual chemical species.

For the latter, our planned hyperspectral evaluation in combination with multivariate analysis will be paramount for this task, as is well known from many examples in the literature of Raman imaging. General overviews for this combination approach may be found e.g. in references [52].

References S.B.3

[46] Monolayer graphene film on quartz - Product Datasheet. Graphenea, San Sebastian, Spain. Online access: https://cdn.shopify.com/s/files/1/0191/2296/files/Graphenea_Monolayer_Graphene_on_Quartz_Datasheet_02-16-2022.pdf?v=1645025384. Last accessed: Dec.10, 2022.

[47] Whitener, Jr., K.E. Review Article – Hydrogenated graphene: A user's guide. *J. Vac. Sci. Technol. A*, **2018**, 36, 05G401.[https://doi.org/10.1116/1.5034433

[48] Fabriciu, A.; Catanzaro, A.; Cultrera, A. (eds.) – The 16NRM01 EMPIR GRACE consortium. Good Practice Guide on the electrical characterisation of graphene using contact methods. EMPIR, Euramet, **2020**. Online access: http://empir.npl.co.uk/grace/wp-content/uploads/sites/32/2020/04/20200428_GRACE_GPG_1_v1.pdf Last accessed: Dec 10,2022

[49] Jiang, L.; Fu, W.; Birdja, Y.Y.; Koper, M.T.M.; Schneider, G.F. Quantum and electrochemical interplays in hydrogenated graphene. *Nature Commun*. **2018**, 9, 793. [https://doi.org/10.1038/s41467-018-03026-0 Last accessed: Dec 10, 2022.

[50] Scardaci, V.; Compagnini, G. Raman spectroscopy investigation of graphene oxide reduction by laser scribing. *Carbon* **2021**, 7, 48. https://doi.org/10.3390/c7020048

[51] Guillemette, J. Electronic transport in hydrogenated graphene. PhD thesis. Department of Physics, McGill University, Montréal, Québec, Canada, **2014**. Online access: https://gervaislab.mcgill.ca/Guillemette_PhD_Thesis.pdf Last accessed: Dec 10, 2022.

[52] Lee, E.; Adar, F.; Whitle, A. Multivariate data processing of spectral images: the ugly, the bad, and the true. *Spectroscopy* **2007**, 22 (8); reprinted by Horiba. Online access: [https://www.horiba.com/fileadmin/uploads/Scientific/Documents/Raman/autumn07.pdf] Last accessed: Dec 10, 2022.
